# Anaplastic Large Cell Lymphoma of the Breast by Race and Ethnicity

**DOI:** 10.1001/jamanetworkopen.2025.28013

**Published:** 2025-09-02

**Authors:** Dylan K. Kim, Lauren S. Lowe, Alfred I. Neugut, James B. Yu, Simon K. Cheng, Lisa A. Kachnic, David P. Horowitz, Christine H. Rohde, Connor Jarrett Kinslow

**Affiliations:** 1 Division of Plastic and Reconstructive Surgery, Department of Surgery, Columbia University Vagelos College of Physicians and Surgeons, Columbia University, New York, New York; 2 Herbert Irving Comprehensive Cancer Center, Columbia University Vagelos College of Physicians and Surgeons and NewYork-Presbyterian, New York; 3 Department of Medicine, Columbia University Vagelos College of Physicians and Surgeons and NewYork-Presbyterian, New York; 4 Department of Epidemiology, Mailman School of Public Health, Columbia University, New York, New York; 5 Smilow Cancer Center at St Francis Hospital, Hartford, Connecticut; 6 Cancer Outcomes, Public Policy, and Effectiveness Research (COPPER) Center at Yale, New Haven, Connecticut; 7 Department of Radiation Oncology, Columbia University Vagelos College of Physicians and Surgeons and NewYork-Presbyterian, New York, New York; 8 Department of Radiation Oncology, James J. Peters Veterans Affairs Medical Center, Bronx, New York; 9 Department of Radiation Oncology, Memorial Sloan Kettering Cancer Center, New York, New York

## Abstract

**Importance:**

The incidence rate of primary breast anaplastic large cell lymphoma (ALCL), a complication associated with breast implants, is rapidly rising in the US. Comprehensive studies on the racial and ethnic epidemiologic characteristics of ALCL in the US are lacking, despite evidence of worldwide geographic variability.

**Objective:**

To characterize the incidence rates of breast ALCL by race and ethnicity in the US.

**Design, Setting, and Participants:**

This retrospective, observational, population-based cohort study obtained data from the Surveillance, Epidemiology, and End Results program database. The cohort comprised women who were newly diagnosed with primary ALCL within the breast between January 1, 2000, and December 31, 2020. Data analysis was conducted from March to June 2024.

**Exposure:**

Patient race and ethnicity.

**Main Outcomes and Measures:**

Age-adjusted incidence rate of breast ALCL per 100 million persons per year.

**Results:**

In a cohort of 868 118 334 women at risk of breast ALCL over 943 941 114 person-years from 2000 to 2020, 90 were diagnosed with breast ALCL and 55 were diagnosed with T-cell lymphoma, not otherwise specified, resulting in 145 women in the combined (breast ALCL plus T-cell lymphoma, not otherwise specified) cohort. The mean (SD) age of this cohort was 57.6 (15.9) years. These patients self-reported as Hispanic (19 [13.1%]) and non-Hispanic American Indian or Alaska Native (<11 [<7.6%]), Asian or Pacific Islander (<11 [<7.6%]), Black (<11 [<7.6%]), and White (105 [72.4%]). The overall incidence rate of breast ALCL was 9.7 (95% CI, 7.7-11.9) per 100 million persons per year. Incidence rates per 100 million persons per year were highest for White (11.6; 95% CI, 9.0-14.9), followed by Hispanic (7.5; 95% CI, 4.0-13.0), Black (3.5; 95% CI, 0.7-10.1), and Asian or Pacific Islander (0.9; 95% CI, 0.0-5.7) patients. From 2000 to 2010 through 2011 to 2020, incidence rates per 100 million persons per year of breast ALCL increased for Hispanic (0.8 [95% CI, 0.0-7.1] to 12.7 [95% CI, 6.5-22.3]) and White patients (3.9 [95% CI, 2.0-6.9] to 20.1 [95% CI, 15.0-26.5]), but no increase was observed for Black patients (4.9 [95% CI, 0.5-17.4] to 2.4 [95% CI, 0.0-12.5]). Similar patterns were observed in a sensitivity analysis incorporating additional cases of T-cell lymphoma, not otherwise specified, to capture upper estimates of incidence rates.

**Conclusions and Relevance:**

In this cohort study, incidence rates of breast ALCL were highest and increased most rapidly in Hispanic and non-Hispanic White patient populations, while only a few cases were reported in American Indian or Alaska Native, Asian or Pacific Islander, and Black populations. Future studies should delineate the factors associated with these differences and continue to monitor textured breast implant utilization.

## Key Points

**Question:**

What are the incidence rates of anaplastic large cell lymphoma (ALCL) of the breast in the US based on race and ethnicity?

**Findings:**

In this cohort study including 90 women with breast ALCL, incidence rates of breast ALCL were highest and increased most rapidly among Hispanic and non-Hispanic White patient populations, while only a few cases were reported in American Indian or Alaska Native, Asian or Pacific Islander, and Black populations from 2000 to 2020.

**Meaning:**

There are notable racial and ethnic differences in the incidence rates of breast ALCL in the US, the reasons for which are not completely known and deserve further investigation.

## Teaser

This cohort study uses data from the Surveillance, Epidemiology, and End Results program to examine the incidence of anaplastic large cell lymphoma of the breast according to the race and ethnicity of US women with this diagnosis.

## Introduction

The incidence rate of primary breast anaplastic large cell lymphoma (ALCL) is rapidly rising in the US and has been associated with the use of textured breast implants.^1,2,3^ Existing literature has described cases of ALK-negative breast ALCL in patients with breast implants, associated with a distinct subtype of ALCL named breast implant–associated ALCL (BIA-ALCL). The incidence rate of breast ALCL was 14 to 19 cases per 100 million persons per year from 2015 to 2018 compared with 3 to 4 cases per 100 million persons per year from 2000 to 2004.^1^ The origin of breast ALCL has not been fully elucidated; however, existing literature suggests that chronic inflammation from breast implants and the creation of a hypoxic microenvironment may be the most well-supported factors in this malignant neoplasm, rather than infection by a bacterial biofilm as previously reported.^4,5,6,7^ Anecdotally, fewer cases of breast ALCL have been reported in Black and Asian or Pacific Islander patients compared with White patients. For example, in PROFILE (Patient Registry and Outcomes for Breast Implants and Anaplastic Large Cell Lymphoma Etiology and Epidemiology), a national US patient registry, of all 162 cases of BIA-ALCL for whom race was known, 1 was identified as Black, 160 as White, and 1 as multiracial.^8^ Furthermore, global incidence rates of breast ALCL vary greatly, with higher incidence rates in the US and Europe and lower incidence rates in other continents, such as Asia and South America.^9,10^ For example, in 2019, Collett et al^10^ described 502 cases in the US and Europe, 19 cases in South America, 2 cases in Asia, and 2 cases in Africa out of 656 total reported cases of BIA-ALCL. It is unknown if racial, ethnic, and geographic variability is due to genetic or lifestyle differences, patterns in breast implant use, or other biases related to the reporting of data.

Variations in global incidence rates may also reflect differences in national reporting systems, which depend on the establishment of cancer registries, active vs passive data collection, and government regulatory agency monitoring.^10,11^ Comprehensive studies on the racial and ethnic epidemiologic characteristics of ALCL in the US are lacking despite evidence of racial and ethnic variability. We therefore sought to characterize the incidence rates of breast ALCL by race and ethnicity in the US.

## Methods

This cohort study was deemed exempt from review and the informed consent requirement by the Columbia University Institutional Review Board based on institutional policy for non–human participant research. This study followed the Strengthening the Reporting of Observational Studies in Epidemiology (STROBE) reporting guideline.

We used the Surveillance, Epidemiology, and End Results (SEER) Program, the National Cancer Institute’s authoritative source for cancer incidence and survival.^12^ Specifically, we derived incidence rates from the SEER 17 database, which includes data from population-based cancer registries in 13 states, covering approximately 27% of the US population.^12,13,14^ All hospitals are mandated by law to report newly diagnosed cancer cases to their state registry.^15^ We, therefore, expected our case ascertainment rate to approach 100%. The National Cancer Institute applies rigorous quality-control checks, including data completeness evaluations and data quality assessments in compliance with standards in the North American Association of Central Cancer Registries. SEER oversamples certain racial and ethnic minority groups to improve diverse population representativeness, including those that are traditionally underserved.^12^ Race is abstracted from medical records, which have a high sensitivity and a positive predictive value for self-reported race.^16,17^ Hispanic or Latino ethnicity is determined according to best practices developed by the North American Association of Central Cancer Registries using both direct (medical records) and indirect methods (algorithm based on race, surname, maiden name, birthplace, and county of residence).^17^

### Statistical Analysis

We queried the SEER 17 database (November 2022 submission, including data from 2000 to 2020) for women newly diagnosed with primary ALCL within the breast between January 1, 2000, and December 31, 2020. Primary ALCL was identified using the *International Classification of Diseases for Oncology*, *Third Revision* (*ICD-O-3*) code 9714, and diagnoses were included only if microscopically confirmed. Age-adjusted incidence was calculated by the direct method to the 2000 US standard population using 5-year age categories. Incidence rates were also compared across the 2 decades of the study period (2000-2010 and 2011-2020). Ten-year periods were selected for this analysis to preserve adequate sample sizes. Rates were described graphically with linear regression models, as described in previous work describing patterns in ALCL.^1^ Incidence rates were stratified by ethnicity as Hispanic or non-Hispanic. Non-Hispanic ethnicity was further specified by race into the following standard SEER categories: American Indian or Alaska Native, Asian or Pacific Islander, Black, and White. For all groups, cell counts were suppressed if counts were fewer than 11 to protect patient privacy.

We also conducted a sensitivity analysis that included additional cases of mature T-cell lymphoma, not otherwise specified (*ICD-O-3* code 9702) into the cohort of interest. Previous work has shown that the addition of this latter code may adequately represent the upper estimate of true breast ALCL incidence.^1,18^ For comparison, the incidence rates of implant-based breast reconstruction and all lymphomas were also calculated and stratified by decade as well as by race and ethnicity. Statistical analyses were conducted from March to June 2024 using SEER*Stat, version 8.3.9 (National Cancer Institute).

## Results

In a cohort of 868 118 334 women at risk over 943 941 114 person-years from 2000 to 2020, 90 women with a diagnosis of breast ALCL were identified. Additionally, 55 women were diagnosed with T-cell lymphoma, not otherwise specified, resulting in 145 women in the combined (breast ALCL plus T-cell lymphoma, not otherwise specified) cohort (Table 1). In the combined cohort, the most common age group was 50 to 59 years (38 [26.2%]), and the mean (SD) age was 57.6 (15.9) years. These patients self-reported as Hispanic (19 [13.1%]) and non-Hispanic American Indian or Alaska Native (<11 [<7.6%]), Asian or Pacific Islander (<11 [<7.6%]), Black (<11 [<7.6%]), and White (105 [72.4%]). The majority of neoplasms (91 [62.8%]) were classified as localized in stage, according to SEER combined staging. Over the study period, 54 496 cancer-directed implant-based breast reconstruction cases were recorded, with non-Hispanic White (hereafter White) patients accounting for the majority of cases (40 063 [73.5%]) (eFigure in Supplement 1).

**Table 1.** Characteristics of All Patients With Breast Anaplastic Large Cell Lymphoma (ALCL) or ALCL Plus T-Cell Lymphoma, Not Otherwise Specified From 2000 to 2020

Variable	Patients, No. (%)	
	ALCL (n = 90)	ALCL plus T-cell lymphoma, not otherwise specified (n = 145)
Age, y		
≥70	15 (16.7)	31 (21.4)
60-69	19 (21.1)	32 (22.1)
50-59	27 (30.0)	38 (26.2)
40-49	18 (20.0)	28 (19.3)
<40	11 (12.2)	16 (11.0)
Year of diagnosis		
2000-2005	<11 (12.2)	14 (9.7)
2006-2010	<11 (12.2)	17 (11.7)
2011-2015	30 (33.3)	43 (29.7)
2016-2020	>39 (>43.3)	71 (49.0)
Stage		
Localized	>52 (>57.8)	91 (62.8)
Regional	<11 (<12.2)	12 (8.3)
Distant	<11 (<12.2)	16 (11.0)
Unknown	17 (18.9)	26 (17.9)
Surgical excision	61 (67.8)	96 (66.2)
Adjuvant systemic therapy	<11 (<12.2)	15 (10.4)
Adjuvant radiation	<11 (<12.2)	12 (8.3)
History of cancer	37 (41.1)	60 (41.4)

The incidence rates of breast ALCL and ALCL plus T-cell lymphoma, not otherwise specified were 9.7 (95% CI, 7.7-11.9) and 15.4 (95% CI, 12.9-18.1) per 100 million persons per year, respectively. The incidence rate of breast ALCL increased over the study period from 3.3 (95% CI, 1.8-5.4) per 100 million persons per year in 2000 to 2010 to 15.9 (95% CI, 12.4-20.0) per 100 million persons per year during 2011 to 2020, and the incidence rate of breast ALCL plus T-cell lymphoma, not otherwise specified increased from 6.7 (95% CI, 4.5-9.5) per 100 million persons per year in 2000 to 2010 to 23.8 (95% CI, 19.5-28.7) per 100 million persons per year in 2011 to 2020 (Figure). When stratified by race and ethnicity, the 2000 to 2020 incidence rates per 100 million persons per year for breast ALCL were 0.9 (95% CI, 0.0-5.7) for Asian or Pacific Islander patients, 3.5 (95% CI, 0.7-10.1) for Black patients, 7.5 (95% CI, 4.0-13.0) for Hispanic patients, and 11.6 (95% CI, 9.0-14.9) for White patients (Table 2). For ALCL plus T-cell lymphoma, not otherwise specified, the corresponding incidence rates per 100 million persons per year were 24.8 (95% CI, 0.6-118.2) for American Indian or Alaska Native patients, 4.7 (95% CI, 1.5-11.4) for Asian or Pacific Islander patients, 10.2 (95% CI, 4.6-19.3) for Black patients, 11.2 (95% CI, 6.7-17.6) for Hispanic patients, and 17.5 (95% CI, 14.2-21.4) for White patients (Table 3).

**Figure.**
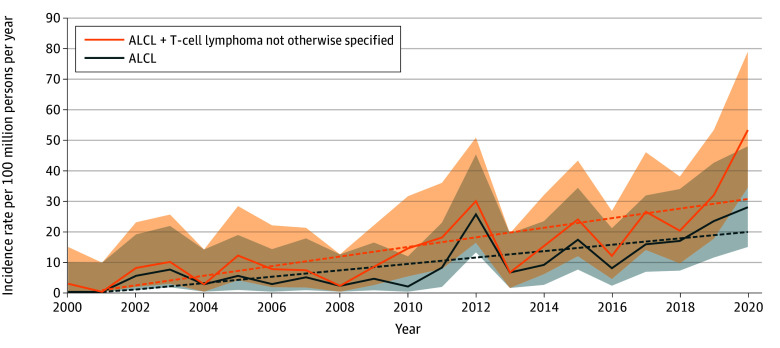
Overall Incidence of Breast Anaplastic Large Cell Lymphoma (ALCL) and Breast ALCL Plus T-Cell Lymphoma, Not Otherwise Specified From 2000 to 2020^a^ Shaded areas indicate 95% CIs, and dotted lines indicate linear regression patterns for each category. ^a^Incidence rates were derived from the Surveillance, Epidemiology, and End Results Program 17 database and adjusted for age.

**Table 2.** Incidence of Breast Anaplastic Large Cell Lymphoma by Race and Ethnicity From 2000 to 2020, Stratified by Decade

Race and ethnicity	Incidence rate per 100 million persons per year (95% CI)^a^					
	2000-2020		2000-2010		2011-2020	
	Cases	Incidence	Cases	Incidence	Cases	Incidence
Hispanic, all races	13	7.5 (4.0-13.0)	<11	0.8 (0.0-7.1)	>2	12.7 (6.5-22.3)
Non-Hispanic American Indian or Alaska Native	<11	NR^b^	<11	NR^b^	<11	NR^b^
Non-Hispanic Asian or Pacific Islander	<11	0.9 (0.0-5.7)	<11	NR^b^	<11	1.7 (0.0-10.7)
Non-Hispanic Black	<11	3.5 (0.7-10.1)	<11	4.9 (0.5-17.4)	<11	2.4 (0.0-12.5)
Non-Hispanic White	69	11.6 (9.0-14.9)	12	3.9 (2.0-6.9)	57	20.1 (15.0-26.5)

Abbreviation: NR, not reported.

^a^ Incidence rates were derived from the Surveillance, Epidemiology, and End Results Program 17 database, from 2000 to 2020, and adjusted for age.

^b^ Incidence rates were not calculated due to an insufficient number of cases.

**Table 3.** Incidence of Breast Anaplastic Large Cell Lymphoma Plus T-Cell Lymphoma, Not Otherwise Specified by Race and Ethnicity From 2000 to 2020, Stratified by Decade

Race and ethnicity	Incidence rate per 100 million persons per year (95% CI)^a^					
	2000-2020		2000-2010		2011-2020	
	Cases	Incidence	Cases	Incidence	Cases	Incidence
Hispanic, all races	19	11.2 (6.7-17.6)	<11	1.7 (0.2-8.2)	>8	18.3 (10.6-29.5)
Non-Hispanic American Indian or Alaska Native	<11	24.8 (0.6-118.2)	<11	57.4 (1.5-266.3)	<11	NR^b^
Non-Hispanic Asian or Pacific Islander	<11	4.7 (1.5-11.4)	<11	6.9 (1.4-20.8)	<11	3.3 (0.4-13.0)
Non-Hispanic Black	<11	10.2 (4.6-19.3)	<11	11.4 (3.6-26.8)	<11	9.0 (2.4-22.8)
Non-Hispanic White	105	17.5 (14.2-21.4)	20	6.4 (3.9-10.1)	85	29.7 (23.4-37.3)

Abbreviation: NR, not reported.

^a^ Incidence rates were derived from the Surveillance, Epidemiology, and End Results Program 17 database, from 2000 to 2020, and adjusted for age.

^b^ Incidence rates were not calculated due to an insufficient number of cases.

Compared with 2000 to 2010, the incidence rates of both ALCL (3.9 [95% CI, 2.0-6.9] to 20.1 [95% CI, 15.0-26.5] per 100 million persons per year) and ALCL plus T-cell lymphoma, not otherwise specified (6.4 [95% CI, 3.9-10.1] to 29.7 [95% CI, 23.4-37.3] per 100 million persons per year) increased in 2011 to 2020 for White patients (Table 2 and Table 3). Similarly, the incidence rate for Hispanic patients increased across decades for ALCL (0.8 [95% CI, 0.0-7.1] to 12.7 [95% CI, 6.5-22.3] per 100 million persons per year) and ALCL plus T-cell lymphoma, not otherwise specified (1.7 [95% CI, 0.2-8.2] to 18.3 [95% CI, 10.6-29.5] per 100 million persons per year). Incidence rates per 100 million persons per year for Black patients decreased between decades for ALCL (4.9 [95% CI, 0.5-17.4] to 2.4 [95% CI, 0.0-12.5]) and ALCL plus T-cell lymphoma, not otherwise specified (11.4 [95% CI, 3.6-26.8] to 9.0 [95% CI, 2.4-22.8]), albeit with widely overlapping CIs. Incidence rates per 100 million persons per year for Asian or Pacific Islander patients for ALCL in 2000 to 2010 and American Indian or Alaska Native patients for both ALCL in all periods and ALCL plus T-cell lymphoma, not otherwise specified in 2011 to 2020 could not be calculated due to an insufficient number of cases.

As a control analysis, the incidence rates of all lymphomas and implant-based breast reconstructions in US women were calculated and stratified by race and ethnicity across the 2 decades (eTable in Supplement 1). The incidence rate of all lymphomas was highest for White patients at 30 779 (95% CI, 30 639-30 920) per 100 million persons per year. This incidence rate decreased from 31 083 (95% CI, 30 886-31 282) per 100 million persons per year in 2000 to 2010 to 30 521 (95% CI, 30 320-30 724) per 100 million persons per year in 2011 to 2020. In comparison, the incidence rate of implant-based breast reconstructions was also highest in White patients at 7388 (95% CI, 7314-7463) per 100 million persons per year. This incidence rate increased from 5131 (95% CI, 5046-5216) per 100 million persons per year to 9990 (95% CI, 9861-10 120) per 100 million persons per year during the study period.

## Discussion

In this study, we ascertained the incidence rates of breast ALCL stratified by race and ethnicity across a 20-year period using a population-based database with large geographic coverage in the US, providing evidence of the epidemiologic differences in incidence rates of breast ALCL among racial and ethnic groups. We found that the incidence rate of breast ALCL increased from 3.3 to 15.9 per 100 million persons per year over the past 2 decades. Incidence rates of breast ALCL were highest and increased most rapidly for Hispanic and White patient populations, while only a few cases were reported in American Indian or Alaska Native, Asian or Pacific Islander, and Black populations.

To date, the rise in overall breast ALCL incidence has been associated with the surge in disease awareness, as evidenced by the creation of new plastic surgery guidelines and a black box warning for breast implants from the US Food and Drug Administration (FDA),^3,19^ in addition to an increase in the use of textured breast implants, which have been traditionally associated with lower rates of capsular contracture, an undesired complication that may be associated with substantial pain and deformity and the need for reoperation.^20,21^ More recent literature has challenged this association and found that textured devices do not hold such advantages over smooth devices consistently in either breast reconstruction or breast augmentation.^22,23,24^ Matros et al^25^ demonstrated that textured breast implant use increased from 3.3% of all implant-based reconstructive and cosmetic cases in 2007 to 22.9% in 2017 within a self-reported plastic surgery procedure registry in the US. Due to the median interval from implant placement to ALCL diagnosis, estimated at 10 years, Matros et al^25^ suggested that rates of BIA-ALCL may continue to increase for the next several years despite recent FDA regulations against textured breast implants. This study did not stratify patterns in textured vs smooth breast implant use by patient race and ethnicity.

As a result of this association with breast implant use, the racial and ethnic differences in incidence rates described in this study may play a role in different rates of breast reconstruction and cosmetic procedures. In breast reconstruction, it is becoming increasingly well recognized that women from racial and ethnic minority groups undergo postmastectomy reconstruction less than their White counterparts.^26,27,28,29^ For example, in an analysis of SEER data from 1998 to 2008, patients of racial and ethnic minority groups was shown to have a lower likelihood of postmastectomy breast reconstruction compared with non-Hispanic White patients. Among racial and ethnic minority groups, Hispanic patients had the highest odds of reconstruction, followed by American Indian or Alaska Native, Asian, and Black patients.^30^ Racial and ethnic minority groups were also shown to have a higher likelihood of undergoing autologous rather than implant-based reconstruction.^31^ In an analysis using a large surgical database, all racial and ethnic minority groups were associated with a lower likelihood of implant-based reconstruction compared with White race.^29^ These differences may be a result of several considerations, such as insurance status, socioeconomic status, access to reconstructive services, clinical factors specific to breast reconstruction (eg, patterns of implant selection and decision to undergo the procedure), data reporting biases, and referral patterns.^27^ These differences in postmastectomy implant use may be associated with the different incidence rates of breast ALCL among racial and ethnic groups; incidence rates were notably highest in the White patient population.

Cosmetic breast surgery, particularly breast augmentation, represents a majority of implant-based plastic surgeries and, as a result, is a factor in the overall use of breast implants.^25^ Although there is no known comprehensive assessment of cosmetic breast implant use by race or ethnicity in the existing literature, the American Society of Plastic Surgeons summarized that White patients accounted for 74% of individuals for all recorded cosmetic breast augmentations in 2020, followed by Hispanic patients at 12%, Asian or Pacific Islander patients at 7%, and Black patients at 6%.^32^ This 74% reflects a larger proportion of White race and ethnicity than in the 2020 US Census (58.4%), although Hispanic ethnicity was relatively underrepresented compared with the Census population (19.5%).^33^ Thus, it is plausible that these differences in implant use may be associated with differences in ALCL incidence. The SEER database does not collect information on the reason for use of breast implants; as a result, there is an evident need to further investigate differences in rates of reception for cosmetic breast surgery, particularly augmentation.

The published literature has also hypothesized a genetic factor in breast ALCL. Using a nationwide Dutch pathology registry, de Boer et al^2^ found that genetic factors, such as *BRCA* sequence variation, may be associated with an increased risk of breast ALCL; however, it is unlikely that sequence variations with a relatively small prevalence in the general population have a role in the racial and ethnic differences described in this study. The incidence of all lymphomas was highest in White patients in this analysis, which may be associated with the relatively high incidence rates of breast ALCL in this patient population. However, the incidence rate of all lymphomas changed only marginally across all racial and ethnic groups and even decreased for White patients between the decades. It is, therefore, more likely that patterns of breast implant use contribute to the increased incidence rate of breast ALCL across the period observed in this study.

### Strengths and Limitations

The results of our study add to the growing literature of breast ALCL and introduces novel evidence of the epidemiologic differences in incidence rates stratified by race and ethnicity. Strengths of this study include the use of the well-validated SEER national cancer database with a large geographical coverage for the estimation of the incidence of breast ALCL.

Limitations include our inability to identify the incidence rate of factors associated with breast ALCL beyond cancer-directed implant-based breast reconstruction, including cosmetic breast implant surgery, contralateral implant-based reconstruction, and non–BIA-ALCL. Additionally, the prevalence of all breast implants or textured breast implants could not be estimated for this population within the limits of the SEER database. However, a previous study estimated the number of breast ALCL cases using the same *ICD-O-3* codes in SEER and found the estimates to be similar to the number of BIA-ALCL cases (353 vs 333 cases) described by FDA medical device reports in the same study period.^1^ Use of the *ICD-O-3* code 9702, which may include cancers other than ALCL, may also introduce variability to incidence rate estimates of breast ALCL. However, a previous analysis found that the *ICD-O-3* codes for breast ALCL (9714) and breast ALCL plus T-cell lymphoma, not otherwise specified (9714 and 9702) have a 100% specificity and a 96% sensitivity, respectively, in a tertiary center cancer registry.^18^ As a result, this combination of *ICD-O-3* codes most likely provides a reliable range of estimates for incidence rates of breast ALCL. Calculations of incidence rates of breast ALCL in this study also did not account for length of exposure to breast implants. Nelson et al^34^ demonstrated that the exposure to textured breast implants may increase the risk of BIA-ALCL in a cohort of patients who received implant-based breast reconstruction.

Lastly, the inherent rarity of breast ALCL resulted in the small sample size and wide CIs for incidence rates, particularly for racial and ethnic minority populations with less representation. For example, although incidence rates decreased for both Asian or Pacific Islander and Black patient populations across the 2 decades despite increases in incidence rates of implant-based reconstruction, these rates were contained in widely overlapping CIs, which limited the strength of any conclusions from these findings. We were unable to disaggregate the Asian or Pacific Islander category, as SEER does not disaggregate this variable for population-based calculations. Previous oncological studies have delineated substantial differences in incidence of cancers and disparities in outcomes for Native Hawaiian, Pacific Islander, Southeast Asian, or Chinese and Japanese patient populations.^35,36,37^

## Conclusions

In this population-based cohort study using a cancer registry, the incidence rate of breast ALCL rapidly increased in the US and varied by race and ethnicity. The overall increase in incidence rates was greatest in Hispanic and White patient populations, while only a few cases were reported in American Indian or Alaska Native, Asian or Pacific Islander, and Black populations. Future research should aim to delineate the factors associated with these differences and continue to monitor textured breast implant use in the next decade given the delayed presentation of BIA-ALCL. Future work may also investigate epidemiologic patterns in the incidence of other breast implant–associated malignant neoplasms, such as squamous cell carcinoma and other lymphomas.^38,39,40^

## Notes

Supplement 1. **eTable.** Incidence of Breast ALCL, Implant-Based Reconstructions, and All Lymphomas by Race and Ethnicity for the Investigated Study Period (2000-2020), Stratified by Decade **eFigure.** Flow Diagram of Population at Risk, Total Person-Years, and Implant-Based Breast Reconstruction Cases When Stratified by Race and Ethnicity

Supplement 2.
Data Sharing Statement
