# Supplementary material for: Anaplastic Large Cell Lymphoma of the Breast by Race and Ethnicity
Source: JAMA Netw Open. 2025 Sep 2;8(9):e2528013. doi: 10.1001/jamanetworkopen.2025.28013 (PMC12406062; doi:10.1001/jamanetworkopen.2025.28013)
Supplement: Supplement 1. — eTable. Incidence of Breast ALCL, Implant-Based Reconstructions, and All Lymphomas by Race and Ethnicity for the Investigated Study Period (2000-2020), Stratified by Decade eFigure. Flow Diagram of Population at Risk, Total Person-Years, and Implant-Based Breast Reconstruction Cases When Stratified by Race and Ethnicity [file jamanetwopen-e2528013-s001.pdf]

Supplemental Online Content

Kim DK, Lowe LS, Neugut AI, et al. Anaplastic large cell lymphoma of the breast by race and ethnicity. *JAMA Netw Open*. 2025;8(8):e2528013. doi:10.1001/jamanetworkopen.2025.28013

eTable. Incidence of Breast ALCL, Implant-Based Reconstructions, and All Lymphomas by Race and Ethnicity for the Investigated Study Period (2000-2020), Stratified by Decade

eFigure. Flow Diagram of Population at Risk, Total Person-Years, and Implant-Based Breast Reconstruction Cases When Stratified by Race and Ethnicity

This supplemental material has been provided by the authors to give readers additional information about their work.

© 2025 Kim DK et al. *JAMA Network Open*

eTable. Incidence of Breast ALCL, Implant-Based Reconstructions, and All Lymphomas by Race and Ethnicity for the Investigated Study Period (2000-2020), Stratified by Decade

		Hispanic, all races	NHAIAN	NHAPI	NHB	NHW
**Overall study period (2000-2020)**	ALCL Cases	13	<11	<11	<11	69
	ALCL Incidence[a]	7.5 (4.0-13.0)	NR[b]	0.9 (0.02-5.7)	3.5 (0.7-10.1)	11.6 (9.0-14.9)
	Implant Reconstruction[a] Incidence	3,670 (3,575-3,768)	3,375 (2,962-3,831)	4,220 (4,090-4,352)	4,462 (4,330-4,597)	7,388 (7,314-7,463)
	All Lymphomas[a] Incidence	26,979 (26,688-27,271)	22,716 (21,547-23,931)	18,367 (18,090-18,647)	30,104 (29,746-30,466)	30,779 (30,639-30,920)
**2000-2010**	ALCL Cases	<11	<11	<11	<11	12
	ALCL Incidence[a]	0.8 (0.0-7.1)	NR[b]	NR[b]	4.9 (0.5-17.4)	3.9 (2.0-6.9)
	Implant Reconstruction Incidence[a]	1,977 (1,871-2,088)	2,587 (2,076-3,186)	2,460 (2,315-2,612)	2,233 (2,100-2,372)	5,131 (5,046-5,216)
	All Lymphomas Incidence[a]	26,789 (26,335-27,247)	20,945 (19,273-22,717)	18,502 (18,074-18,938)	29,064 (28,544-29,590)	31,083 (30,886-31,282)
**2011-2020**	ALCL Cases	>2	<11	<11	<11	57
	ALCL Incidence[a]	12.7 (6.5-22.3)	NR[b]	1.7 (0.0-10.7)	2.4 (0.0-12.5)	20.1 (15.0-26.5)
	Implant Reconstruction Incidence[a]	4,995 (4,847-5,146)	4,227 (3,569-4,970)	5,682 (5,479-5,892)	6,572 (6,346-6,803)	9,990 (9,861-10,120)
	All Lymphomas Incidence[a]	27,168 (26,789-27,551)	24,305 (22,671-26,025)	18,343 (17,977-18,714)	31,011 (30,514-31,514)	30,521 (30,320-30,724)

Abbreviations: ALCL, anaplastic large cell lymphoma; NHAIAN, Non-Hispanic American Indian/Alaskan Native; NHAPI, Non-Hispanic Asian/Pacific Islander; NHB, Non-Hispanic Black; NHW, Non-Hispanic White
[a]Incidence rates are derived from the SEER 17 database, 2000-2020, adjusted for age. 95% confidence intervals are also provided in parentheses. Incidence rates are calculated per 100 million persons per year.
[b]Incidence rates were not calculated due to an insufficient number of cases.
NR – Not reported

© 2025 Kim DK et al. *JAMA Network Open*

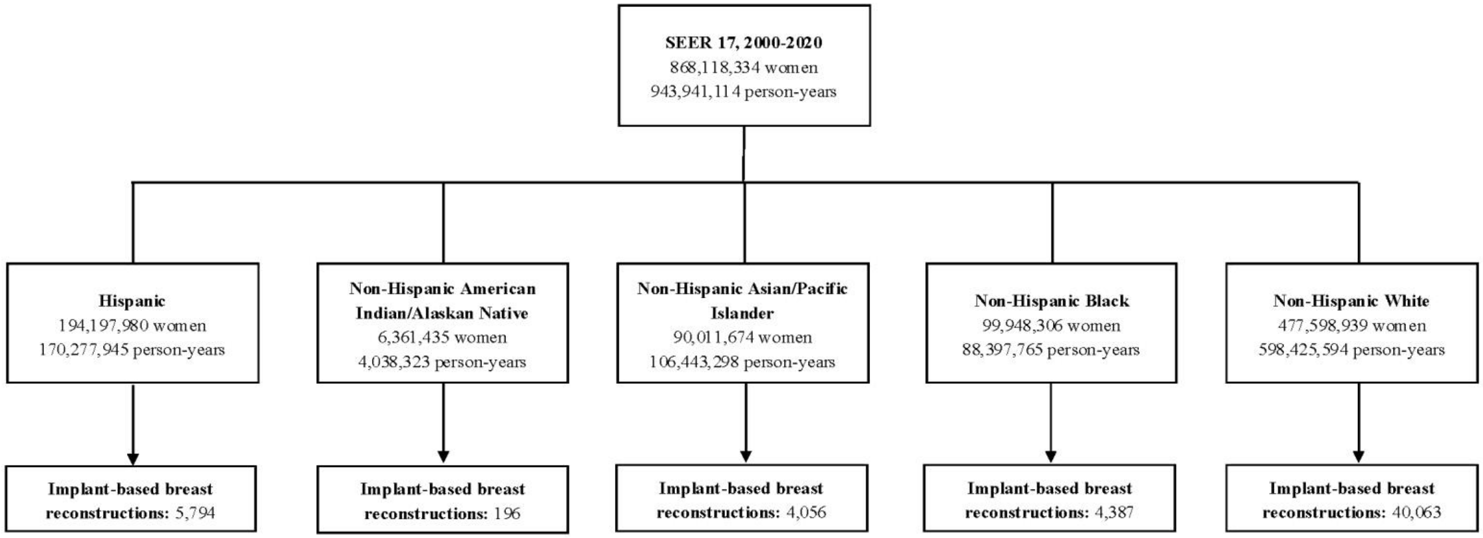

eFigure. Flow Diagram of Population at Risk, Total Person-Years, and Implant-Based Breast Reconstruction Cases When Stratified by Race and Ethnicity

© 2025 Kim DK et al. *JAMA Network Open*
