# Supplementary material for: Anaplastic Large Cell Lymphoma of the Breast by Race and Ethnicity
Source: JAMA Netw Open. 2025 Sep 2;8(9):e2528013. doi: 10.1001/jamanetworkopen.2025.28013 (PMC12406062; doi:10.1001/jamanetworkopen.2025.28013)
Supplement: Supplement 2. — Data Sharing Statement [file jamanetwopen-e2528013-s002.pdf]

Data Sharing Statement

Kim. Anaplastic Large Cell Lymphoma of the Breast by Race and Ethnicity. *JAMA Netw Open*. Published August 21, 2025. doi:10.1001/jamanetworkopen.2025.28013

Data
Data available: No

Additional Information
Explanation for why data not available: The data underlying this study are available upon direct request to the NCI/SEER.
